# Poor Reporting of Outcomes Beyond Accuracy in Point-of-Care Tests for Syphilis: A Call for a Framework

**DOI:** 10.1155/2014/465932

**Published:** 2014-03-27

**Authors:** Yalda Jafari, Mira Johri, Lawrence Joseph, Caroline Vadnais, Nitika Pant Pai

**Affiliations:** ^1^Department of Epidemiology, Biostatistics & Occupational Health, McGill University, Montreal, QC, Canada H3A 1A2; ^2^Department of Health Administration, Université de Montréal, Montreal, QC, Canada H3C 3J7; ^3^Unité de Santé Internationale (USI), Centre de Recherche Hospitalièr de l'Université de Montréal (CRCHUM), Montreal, QC, Canada H2X 0A9; ^4^Division of Clinical Epidemiology, Department of Medicine, Royal Victoria Hospital, McGill University Health Centre, V Building (V2.19), 687 Pine Avenue West, Montreal, QC, Canada H3A 1A1; ^5^Department of Medicine, McGill University, Montreal, QC, Canada H3A 1A1

## Abstract

*Background*. Point-of-care (POC) diagnostics for syphilis can contribute to epidemic control by offering a timely knowledge of serostatus. Although accuracy data on POC syphilis tests have been widely published, few studies have evaluated broader outcomes beyond accuracy that impact patients and health systems. We comprehensively reviewed evidence and reporting of these implementation research outcomes (IROs), and proposed a framework to improve their quality. *Methods*. Three reviewers systematically searched 6 electronic databases from 1980 to 2014 for syphilis POC studies reporting IROs. Data were abstracted and findings synthesised narratively. *Results*. Of 71 studies identified, 38 documented IROs. IROs were subclassified into preference (7), acceptability (15), feasibility (15), barriers and challenges (15), impact (13), and prevalence (23). Using our framework and definitions, a pattern of incomplete documentation, inconsistent definitions, and lack of clarity was identified across all IROs. *Conclusion*. Although POC screening tests for syphilis were generally favourably evaluated across a range of outcomes, the quality of evidence was compromised by inconsistent definitions, poor methodology, and documentation of outcomes. A framework for standardized reporting of outcomes beyond accuracy was proposed and considered a necessary first step towards an effective implementation of these metrics in POC diagnostics research.

## 1. Introduction

Syphilis is an important public health issue; the latest available worldwide estimates conducted by the World Health Organization in 1999 suggested approximately 12 million cases of syphilis [1]. With 90% of infected individuals unaware of their serostatus, a lack of timely diagnosis is a driving force of the syphilis epidemic, particularly in resource limited settings where use of standard diagnostics presents important challenges [2]. Standard syphilis screening involves initial testing with a nontreponemic specific assay and confirmation with a treponemal specific assay [3–6]. These tests are usually expensive, must be conducted by skilled, laboratory-based personnel, and have a long turnaround time resulting in loss of patients to followup [7, 8]. Point-of-care (POC) tests therefore offer a novel, low cost, easy to use solution that provides results in a single visit, expediting linkages to confirmatory testing, treatment, and referral. Designed for settings with limited infrastructure, POC technologies are potentially transformative tools for global syphilis control [3].

Systematic reviews demonstrated the accuracy of syphilis POC tests [9, 10]. However, as advisory bodies on diagnostics have emphasized, accuracy is a necessary but not a sufficient condition for widespread uptake of syphilis POC tests [11]. Our objective is to facilitate decision-making related to introduction of syphilis POC tests for health planners and policymakers in diverse geographical settings, by synthesising evidence on outcomes beyond accuracy, referred to as implementation research outcomes (IRO). These IROs are relevant to patients, caregivers, and health systems. This systematic review presents the first comprehensive portrait of available evidence on IROs for syphilis POC tests.

## 2. Materials and Methods

### 2.1. Information Sources

We searched the MEDLINE, EMBASE, GLOBAL HEALTH, CINAHL, Web of Science, and SCOPUS electronic databases over the period from January 1, 1980, to January 31, 2014. All languages were considered and non-English articles were translated.

#### 2.1.1. Search String

We used the following terms: (syphilis OR* Treponema pallidum*) AND (point-of-care OR rapid test OR rapid assays). Keywords rather than MeSH terms were used to capture recent papers not yet indexed and to be able to search multiple databases at once. No filters for diagnostic studies were applied as these have been shown not to capture all relevant studies [12].

### 2.2. Study Selection

Three reviewers (Yalda Jafari [YJ], Sushmita Shivkumar [SS], and Rohit Vijh [RV]), independently conducted database searches and reviewed the titles and abstracts of articles retrieved. Articles satisfying the eligibility criteria were retained for full-text screening. Study inclusion was determined by discussion between the initial reviewers who, in cases of discordance, appealed to a fourth reviewer (Nitika Pant Pai [NPP]).

### 2.3. Eligibility Criteria

We considered studies satisfying the following criteria.

*Study Design*. All studies conducted on humans or human samples reporting IROs.

*Participants*. Live participants of any age and risk group using whole blood or serum specimens, either from participants or serum panels.

*Interventions*. Studies documenting the use of syphilis POC tests.

*Outcome Measures*. To systematize presentation, documented IROs were tabulated according to the working definitions in a framework proposed below.

- Preference: documented as a proportion (numerator/denominator) with 95% confidence intervals (CI). Numerator was defined as the number of study participants (i.e., patients, doctors, nurses, and lab technicians) in the study interviewed that preferred the POC test or testing strategy to the conventional reference testing strategy. Denominator was defined as the total number of participants (i.e., patients, doctors, nurses, and lab technicians) in whom the new POC test or strategy was evaluated.
- Acceptability: documented as a proportion (numerator/denominator) with 95% CI. Numerator was defined as the number of study participants (i.e., patients, providers, etc.) who accepted the new POC test/strategy over the conventional reference test/strategy. Denominator was defined as the total number of participants that were offered the strategy. Participants who consented to study participation were offered the new test/strategy and acceptability was thereby documented.
- Feasibility: feasibility of test/strategy evaluated in the study was documented. Feasibility of a test/strategy was defined with completion rate of implementation of POC strategy [13]. The completion rate (a misnomer) was documented as a proportion with 95% CI. Completion typically referred to completion of the test or screening procedure. This included screening with a POC test, typically followed by initiation of linkages to improve clinical management of patients. These linkages varied from confirmatory testing of POC test results to receipt of treatment based on POC results or to downstream treatment in referral clinics. Other associated metrics/factors relevant to feasibility of a strategy were patient experience related such as convenience, comfort, noninvasiveness, or ease of use of POC tests (reported as yes/no) and/or time metric related, that is, turnaround time to test results (TAT) with POC versus standard laboratory tests also included. Turnaround time to test results with POC was documented in minutes/hours. This TAT included waiting time to test result. Turnaround time to treatment initiation or time to receipt of confirmatory test results, if reported, was also documented. All time was reported in minutes/hours. Mean time with interquartile ranges or average (median) if available was also documented. Reported barriers and challenges to implementation of POC tests were abstracted and reported sometimes under feasibility.
- Barriers and challenges: reported barriers and challenges to implementation of POCs are recorded.
- Impact: impact was described by its different facets including (1) proportional increase in new cases picked up with the new POC strategy compared to the reference conventional strategy, (2) proportional increase in numbers that received treatment with the new POC strategy over reference conventional strategy, (3) a reduction in time to confirmatory testing with novel POC versus conventional strategy, (4) an increase in the number of sexual partners notified with novel POC test versus reference conventional, (5) documentation of provision of interventions to the mother and infant (as in numbers/proportions) with same day testing and treatment linked with new POC strategy over a conventional strategy, and (6) lastly, a reduction in time to referral linkages with novel POC versus conventional strategy. Data permitting, proportions were expressed with 95% CI.
- Prevalence: study prevalence (typically, period prevalence and, sometimes, point prevalence) was defined as a proportion, where numerator consisted of individuals who tested seropositive during the study period, and denominator consisted of the total study sample [14]. If available, 95% CI was expressed. For the purposes of the review, studies that used POC tests solely to determine prevalence of syphilis in the study sample were included in this category.

### 2.4. Data Extraction

Reviewers extracted relevant information independently using a standardized data abstraction form pretested on a subset of the sample. One reviewer (YJ) extracted data on 100% of papers, and second reviewers (SS & RJ) abstracted data on 50% of papers.

### 2.5. Summary Measures

A narrative approach was taken to synthesize outcome data. This is standard practice for systematic reviews with considerable methodological heterogeneity such as IROs.

## 3. Results

**Figure 1:**
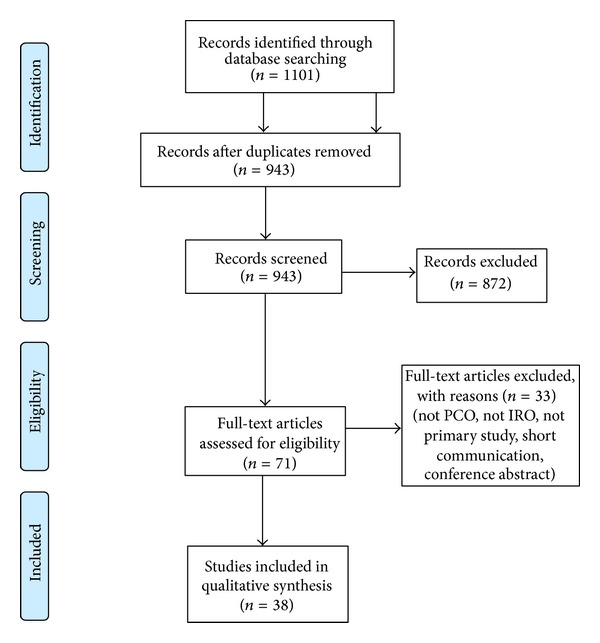
Study selection following PRISMA guidelines.

Of 71 full-text articles, 38 (53.5%) documented IROs. Following PRISMA guidelines [15], our study selection process has been presented in Figure 1. Of 38 articles, three studies (7.9%) were in languages other than English (2 in Spanish, 1 in Portuguese) and were translated. Thirty-four (89.5%) articles referred to studies conducted in low- and middle-income country settings. Thirty-four studies employed a cross-sectional design (89.5%), one was a case-control (2.6%), and one was a clustered randomized trial (2.6%) design. Study descriptions for IROs are available in Table 1.

- Preference: seven articles discussed preference for POC strategies as compared to conventional strategies [16–22]. In a Brazilian study [16], 60% of clinicians and 52% of patients preferred conventional testing over the POC strategy. Other studies documented preference for the POC strategy amongst nurses (68% [17]) and patients (62% [19], 66.1% [23]) over the conventional approach. In a study conducted in India, 99.3% (95% CI: 98.8, 99.8) of patients preferred testing with 3 POC tests simultaneously to detect syphilis, HIV, and hepatitis B [20]. In an Australian study [18], 79% preferred self-testing with a POC test. In a multicountry study [22] most clients expressed preference for finger prick rather than venipuncture.
- Acceptability: fifteen studies reported on patient and provider acceptability of POC tests [16, 17, 20, 22–33]. The POC testing strategy was found to be highly acceptable to patients [16, 20, 23, 24, 26–29, 31–34], clinicians [17, 22, 26], and lab technicians [16, 26].
- Feasibility: fifteen articles reported on the feasibility of using POC tests and test performance characteristics [4, 16–20, 22, 23, 25, 32, 34–38]. Completion rate of POC testing was high in two studies [20, 23], as patient and provider satisfaction were reported with POC testing in one study [38]. Clinicians and laboratory staff found use and interpretation of results easy [4, 17, 19, 22, 35, 37]. In six studies, a reduction in turnaround time to testing and obtaining results was well received [16, 18, 19, 23, 35, 38]. In Brazil, 90% of patients reported that they would wait up to 30 minutes for results [35]. Simultaneous testing with 3 singleton POC tests (i.e., syphilis, HIV, and hepatitis B) was conducted in India and a TAT of 25 minutes (range 21–27 minutes) was reported [20]. Similarly, in a study conducted in South Africa, a TAT of 15–20 minutes was reported [36]. In three other studies, patients experienced low pain and discomfort with a finger prick, in comparison to tests requiring venipuncture: 77% [38], 91.1% [18], and 68% [16]. In a multicountry study, almost all clients who received a positive POC result received treatment, ranging from 93.6% in Brazil to 103.6% in Tanzania [22]. In an article describing studies in Uganda and Zambia with a POC strategy, almost all participants (99.0% in Uganda and 95.8% in Zambia) received same day testing and treatment with the novel POC strategy [32]. Additionally, partner testing for syphilis was also performed on-site, thereby proving feasibility of the strategy [32].
- Barriers and challenges: barriers and challenges hindered an ideal implementation of POC testing strategies and impeded feasibility. Fifteen studies provided qualitative discussions of these challenges [16–20, 23, 26, 27, 29, 34, 35, 37–40]. From the patient perspective, assumed pain from finger prick was a hindrance to testing [19, 23, 29], as were reports of pain or discomfort [16, 20, 40]. Patients also expressed a distrust of POC test results [19]. From the providers' perspective, challenges included a lack of confidence in POC test results due to the inability to differentiate between past and present infections leading to overtreatment [16, 17, 19, 35]. In addition, disagreements between test readers [37], suboptimal field conditions [34], unreadable [36] or difficulty reading results [39], concerns over procurement of POC tests [38], and the time-consuming nature of offering one-stop services with POC [19, 38] posed challenges in delivery of these services. Furthermore, not all patients agreed to undergo confirmation testing [27], hampering ideal execution of testing process. In two articles evaluating POC tests in Mongolia, inadequacies of partner notification strategies for women were brought to light, raising issues such as risk of reinfection of women if partners are not treated [40] and risk of partner violence [40]. A lack of political leadership, required to ensure the success of antenatal care (ANC) programs to prevent congenital syphilis, was also highlighted as a major barrier to uptake of POC testing [26].
- Impact: fourteen studies reported on the impact highlighting increased testing and treatment of study participants with the implementation of a novel POC screening strategy in comparison to the reference conventional screening strategy [17, 20, 22, 27, 29, 30, 32, 36, 39–44]. A study in Mongolia measured impact of POC tests in a cluster randomized trial design [40]. In comparison to clinics that used conventional testing, clinics with POC testing showed a significantly increased number of women who were tested and a significantly increased number of women and their partners who were treated [40]. In a multicountry study, the increase in numbers screened compared to preimplementation of POC testing ranged from 1.0% in China to 9.2% in Zambia [22].
- Prevalence: twenty-three articles [18, 20, 21, 23–28, 30–32, 39, 41, 44–52] documented prevalence in various populations and estimated the seropositivity in the study sample. Prevalence estimates varied in populations and regions and varied with samples and risk profile of participants. In ANC attendants, prevalence ranged from 0% in Afghanistan [51] to 9.2% in Zambia [32]. In high-risk populations, the prevalence ranged from 0% in Guatemala [48] to 8.9% in eastern Sudan [46].

## 4. Discussion

Using our framework, all the studies could be evaluated for quality of their reporting. Although evidence was primarily obtained from cross-sectional studies, outcomes were incompletely defined and reported in many studies. IROs such as preference, acceptability, feasibility, barriers and challenges, and impact outcomes were not well defined. Furthermore, the quality of evidence was impaired by unclear definitions/framework for IROs for diagnostic studies. Documentation was unclear and IROs were often expressed as percentages often without confidence intervals or without a denominator. Heterogeneity of definitions, lack of reporting of study methodology, and poor quality of reporting hampered drawing of meaningful results, although the bulk of evidence was reported in favour of POC strategies.

A determined effort needs to be made to improve the quality of assessment and reporting of IROs beyond accuracy. Impact outcomes have been clearly defined by the International Initiative for Impact Evaluation [53]. Often a vast majority of studies are cross-sectional and conducted to evaluate the feasibility of a POC strategy. Patient centered outcomes of acceptability, preference, and patient experience become equally important to justify the benefits of an alternative POC strategy to stakeholders or to scale up a project once feasibility is proven. A framework for reporting outcomes that has been proposed and utilized in this review could then be used. Clear definitions and transparency of IROs will not only help improve reporting of studies and quality of research with standardized reporting, but also facilitate comparability of findings for evaluation, across all settings. Standardized reporting will allow policymakers and providers to draw meaningful conclusions for their practice and policy. Reporting results with 95% CIs should also be emphasized. These help understand and appreciate the range of variability possible for an outcome. With these improvements, research on POC diagnostics beyond accuracy will not only be more meaningful but will also positively impact public health and clinical practice [54, 55].

## 5. Conclusion

In view of the growing interest in the elimination of mother to child transmission of HIV and syphilis promoted by WHO and UNICEF and an ever growing need for simultaneous rapid screening tests for HIV and related coinfections for at risk populations, POC screening strategies stand to become more complex in the near future. To report outcomes and metrics, we need to standardize and emphasize precise reporting, documentation, and measurement in publications. In this paper, we have presented a framework that defines a few of the commonly reported IROs beyond accuracy in research that involves POCs in syphilis. To improve evidence on metrics, a methodological framework that standardizes measures and parameters and ensures standardized collection, reporting, and documentation of data beyond accuracy must be emphasized. This move will greatly improve the quality of outcomes reporting in POC diagnostics and allow for enhanced documentation beyond traditional “accuracy outcomes.”

## 6. Summary

Implementation research outcomes documented in point-of-care (POC) diagnostic evaluations for syphilis are poorly defined, evaluated, and reported across studies. We defined operational definitions and proposed a framework to help evaluate the reporting of these outcomes. This framework for syphilis POC diagnostics research will facilitate standardized collection, documentation, synthesis of outcomes, and their inclusion in policy initiatives.

## Figures and Tables

**Table 1:** Description of studies.

Study	Setting	Population and sample size	Index test	Study design	IRO preference	IRO acceptability	IRO feasibility	IRO barriers and challenges	IRO impact	IRO prevalence
Amadi et al., 2010 [45]	Nigeria, urban (LM)	100 dental clinic patients	Syphilis Ultra Rapid Test Strip	CS						1%

Benzaken et al., 2007 [35]	Brazil, urban (UM)	Study 1: 541 STD clinic attendees. Study 2: 248 STD clinic attendees	Study 1: SD Bioline Syphilis and Syphicheck-WB Study 2: VisiTect Syphilis and Determine.	CS			100% of professionals rated instructions and results interpretation as easy. Study 1: reproducibility between clinic and lab professionals had agreement of 99% and kappa index >0.95% for both tests. Study 2: agreement between clinic and lab professionals was 100%. In both studies, 100% of participants were willing to wait up to 30 minutes for their results.	Unnecessary treatment was an issue.		

Benzaken et al., 2008 [19]	Brazil, urban (UM)	510 STI clinic attendees including MSW and FSW; operational characteristics were collected from 12 clinic staff and 60 clients.	Visitect	CS	Clients: 62% preferred POC strategy.		Clinic staff: 75% found test easy to use and 67% found the test easy to interpret. Clients: 93% were not deterred by the waiting time.	Clients: waiting time (7%), cost of transport (10%), opening hours (25%) or lack of trust in POC test results (3%), pain caused by finger prick (57%), and preference for venous blood (38%) collection were some barriers. Clinic staff: only half trusted its results, mostly cited reason being due to inability of the test to differentiate between old and recent syphilis leading to overtreatment and also experienced frequent discordant results between POC and confirmatory tests.		

Bronzan et al., 2007 [17]	South Africa, rural (UM)	1285 ANC attendees.	Determine	CS	Nurses: 68.2% preferred on-site ICS over on-site RPR and off-site RPP/TPHA.	Nurses: on-site testing was acceptable and favoured over off-site testing as it allowed for prompt diagnosis, patient education, and immediate treatment.	100% found on-site ICS test easy to perform, fast, and reliable, compared to 95% who found on-site RPR time consuming, unreliable, and difficult to perform and read.	Unnecessary treatment.	89.4% of women with high-titer syphilis received treatment with ICS versus 63.9% in women tested with on-site RPR and 60.8% tested with off-site approach.	

Callegari et al., 2014 [24]	Brazil, urban (UM)	438 adult patents (18 years plus) of outpatient clinic.	Rapid check syphilis immunochromatographic treponemal test.	CS						5.3% (95% CI: 3.3–7.3)

Campos et al., 2006 [34]	Peru, urban (UM)	3586 FSW in commercial sex venues.	Determine	CS		97.4% agreed to POC test.	Easy to adapt, implement, and integrate with existing into existing work-based STI prevention services.	Inadequate lighting in the field was a problem.		

Chen et al., 2013 [25]	China, urban (LM)	1808 FSW	Wantai Anti-TP Antibody Rapid Test (RST).	CS		94.2% (95% CI 93.1–95.2%) got the testing.	95.2% (95% CI 94.1–96.1%) were willing to get RST on-site.			8.5%

Chen et al., 2012 [23]	China, urban (LM)	2812 FSW	Wantai Anti-TP Antibody Rapid Test.	CS	66.1% preferred finger prick over blood draw.	95.0% (95% CI: 94.1–95.7%) accepted rapid test.	99.3% (95% CI: 98.9–99.5%) of those accepted testing got the test. 52.5% preferred verbal on-site method of result notification.	57.7% listed pain as reason for not wanting to be finger pricked.		6.8%

Dayan et al., 2013 [21]	Turkey, urban (UM)	266,035 healthy blood donor samples.	Architect (Abbott).	Retrospective cohort.						0.07

Dlamini et al., 2014 [36]	South Africa, urban and rural (UM).	297 samples from different health facilities.	SD Bioline, Hexagon	CS			Testing with both tests was completed within the recommended 15–20 min.		3.4% of SD Bioline results were unreadable. Visibility was improved on repeat testing. Hexagon results were more clear and quick in comparison to SD Bioline.	

Elhadi et al, 2013 [46]	Sudan, urban (LM)	4220 FSW	SD Bioline	CS						Ranged from 1.5% in the northern zone to 8.9% in the eastern zone (of their division of Sudan).

Garcia et al., 2013 [39]	Peru, urban (UM)	17,155 ANC attendees.	SD Bioline	CS				9 of 604 (44.5%) health providers participating in the training had difficulties with near vision which did not allow them to recognize the line in the POCT" and 0.3% were colour blind and could not see the red line.	Improved treatment coverage with 91.6% receiving at least one dose of penicillin and 80% with two doses reduced a process that consisted of 27 days in 6 visits to 1 visit in one day; total screening coverage was 94.8%.	0.90%

García et al., 2007 [26]	Bolivia, urban and rural (LM)	11,618 ANC attendants.	Determine	CS		POC testing highly acceptable to participants, clinicians, and laboratory technicians.		Political instability; difficult to ensure continuity.		5%

Gupte et al., 2011 [27]	India, urban (LM)	19,809 female, male, and transgender sex workers.	Syphicheck-WB	CS		Acceptance was high, ranging from 76.0% in MSW to 57.1% bar-based FSW.		Only 2/3 of those positive with POC agreed to RPR confirmation.	Using POC, during 3-month intervention period, syphilis test uptake was 63.1%, more than 4-fold higher than the monthly average uptake of 14.3% at clinic sites during the preceding 9-month period.	Life time infection 3% active syphilis confirmed with RPR-1.2%.

Herring et al., 2006 [4]	South Africa (UM), Gambia (L), Tanzania (LI), China (LM), Sri Lanka (LM), Haiti (L), USA (H), Russian Federation (UM).	Evaluation panel from archived specimens.	Determine Syphilis Fast Espline TP Syphicheck-WB SD Bioline Visitect Syphilis.	CC			Multisite evaluation six kits scored on clarity of instructions, technical complexity, ease of interpretation, and equipment required but not provided. Determine scored the highest and Syphilis Fast scored the lowest. Test reproducibility variability was low.			

Hurtado et al., 2010 [47]	Spain, urban (H)	500 MSM in saunas and flats.	Determine	CS						5% in saunas. 2.3% in apartments.

Juárez-Figueroa et al., 2007 [37]	Mexico, urban (UM)	548 FSW and women within 24 h postpartum.	Determine	CS			All 3 readers found POC test highly user-friendly, and there was agreement in 544 of 548 results (99.3%).	Disagreements were due to appearance of faint lines.		

Lahuerta et al., 2011 [48]	Guatemala, urban (LM)	2874 FSW, MSM, TG MSM, NR (MV: 1336, STI clinic: 1538).	Determine	CS						MV: NR: 0.8%, MSM/TG: 0%, FSW: 4.6%. STI clinic: NR: 0.6%, MSM/TG: 1.4%, FSW: 1.3% .

Lee et al., 2010 [18]	Australia, urban (H)	183 MSM	Determine	CS	79% preferred rapid testing at clinic to venipuncture and serology. 54% preferred self-test if it was available.		Of those who preferred POC at clinic over venipuncture and serology, reasons were immediacy of the result (32.6%), reduced pain or invasiveness (8.9%), and the convenience of not requiring a second clinic visit for test results (4.4%).			3.8%

Mabey et al., 2012 [22]	Brazil, rural (UM) China, rural Peru, rural and urban (UM) Tanzania, rural (L) Uganda, rural (L) Zambia, rural and urban (LM).	Over 100,000 ANC attendees.	China: Rapid Syphilis test Rest: SD Bioline Rapid Syphilis test.	CS	Most clients preferred a finger prick over venipuncture because of the smaller volume of blood required.	POC tests were well accepted by health care workers.	All health care workers in all sites thought POC tests were easy to perform. For instance, 82% of Ugandan health care workers reported POC tests as “very easy to perform.” Clients liked receiving results and treatment on the same day as testing rather than having to return. Almost all who tested positive received treatment: 100% in Brazil, 93.6% in China, 97% in Peru, 90.1% in Tanzania, 103.6% in Uganda, and 95.2% in Zambia.		Change in percent of population that were screened following a POCT introduction was 1.6% in Brazil's sexually active population and 1.4% in their ANC population. Increase in screening was seen in other sites: 1.9% in China, 1.0% in Peru, 10.9% in Tanzania, 5.3% in Uganda, and 9.2% in Zambia.	

Manavi et al., 2012 [28]	UK, urban (H)	405 men attending gay pride event.	OraSure assay	CS		96% accepted testing.				0.5%

Miranda et al., 2009 [41]	Brazil, urban (UM)	1380 ANC attendees.	Determine	CS					5.1% with no previous prenatal care learned serostatus among which 1 was positive detected.	0.4%

Mishra et al., 2010 [29]	India, urban (LM)	4871 FSW attending STI clinic.	Qualpro Syphicheck.	CS		Of the 4157 first-time attendees offered testing, 1117 (26.9%) accepted.		3% of refusals were due to unwillingness to undergo a finger prick due to perceived pain, discomfort, or other.	Acceptance of syphilis increase significantly by 8.0% during the POC protocol versus the standard. Compared to a historical comparison, POC protocol allowed for significantly increased treatment coverage at 16.4%.	

Munkhuu et al., 2009 [40]	Mongolia, urban (LM)	3850 ANC attendees in intervention and 3850 in control group.	SD Bioline Syphilis.	CRT				Some women received unnecessary treatment. Some partners did not receive treatment; thus women were left at risk of reinfection.	Significantly higher number of women at intervention clinics than at control clinics were tested for syphilis at 1st visit (99% versus 79.6%). Similar result was found at 3rd trimester visit. As well, significantly higher number of infected women and their partners were treated at intervention clinics while lower number of congenital syphilis cases occurred.	

Munkhuu et al., 2009 [38]	Mongolia, urban (LM)	246 ANC attendants	SD Bioline Syphilis	CS			All clients preferred receiving results the same day. Women were well satisfied with POC testing. Some cited reasons were time savings (88%), rapid results and no pain (77%), and counselling (42%).	Risk of intimate partner violence in case of discordant results between patient and husband. Time consuming as provider is still expected to complete their “regular” tasks. Concerns over procurement of POC tests.		

Onwuezobe et al., 2011 [49]	Nigeria, urban (LM)	415 ANC attendees.	ACON Ultra Rapid Syphilis Test Strip.	CS						2.2%

Pai et al., 2012 [20]	India, rural (LM)	1066 ANC attendees	Determine	CS	99.3% (95% CI: 98.8, 99.8) preferred 3-in-1 testing to conventional strategies.	98% consented to testing.	96% completed study procedure. Time it took for STPOC was 25 min (range 21–27) versus 45 min (range 40–47) for strategy.	32.1% (95% CI: 29.2–35.0%) reported prick pain.	In the same visit, women were tested and all positives were treated, unlike the traditional method.	0.2% (95% CI: 0–0.48)

Parthasarathy et al., 2013 [42]	India, urban and rural (LM)	330,000 FSW, 82,000 MSM, 10,000 IDU.	Immunochromatographic strip test (ICST) used from 2007 (study conducted from 2004 to 2007).	Retrospective analysis					In comparison to RPR testing, ICST used for screening increased from 7.4% in 2007 to 77.0% (*P* < 0.001) in 2009. During the same period, the syphilis screening rates among clinic attendees increased from 9.0% to 21.6% (*P* < 0.001).	

Read et al., 2013 [30]	Australia, urban (H)	98 MSM attending testing tent at a fair	Determine	CS		Participating rate of 0.23% (2010) and 0.26% (2011).			All testing tent clients were successfully contacted and treated and undertook partner notification.	1.02% (95% CI: 0.03–5.55)

Revollo et al., 2007 [50]	Bolivia, urban (LM)	1594 postpartum women in hospital.	Determine	CS						7.2% (95% CI: 6.5–7.9)

Sabidó et al., 2009 [16]	Brazil, urban (UM)	60 high risk populations, 10 clinical and 2 lab staff.	Visitect Syphilis test.	CS	60% of clinical staff and 52% of clients preferred conventional over POC testing.	Acceptable to patient and laboratory technicians.	Staff: 9/12 found test instructions easy/very easy. Clients: 69% of clients found time waiting for testing as short. 68% found that POC did not cause any discomfort. 95% of patients would recommend this test to others.	6/10 clinical staff lacked confidence in POC results.		

Seguy et al., 2008 [31]	Guyana, rural (LM)	5618 miners	Determine	CS		80% of miners approached accepted testing.				6.4% (95% CI: 4.5, 9.1)

Smit et al., 2013 [43]	Tanzania, urban and rural (L).	2099 ANC attendees.	SD Bioline	CS					If POC testing is in ANC clinics, 82% of pregnant women would receive testing and treatment, compared to 16% if no POC is used.	

Strasser et al., 2012 [32]	Uganda, rural and urban (L) Zambia, rural and urban (LM).	Uganda: 14,540 ANC attendees; Zambia: 11,985 ANC attendees.	SD Bioline	CS study, with pre-post intervention design and retrospective review.		Uganda: 90.3% tested. Zambia: 95.6% tested.	Uganda: 99.0% of those treated received STAT 9.9% of partners tested for syphilis. Zambia: 95.8% of those treated received STAT 3.0% of partners tested for syphilis.		Significantly higher number of women tested and treated and their partners tested using POC intervention versus the traditional method.	Uganda: 5.3%. Zambia: 9.2%.

Todd et al., 2011 [52]	Afghanistan, urban (L).	483 IDU.	SD Bioline	CS						2.1% (95% CI: 1.0–3.8)

Todd et al., 2008 [51]	Afghanistan, urban (L).	4452 ANC attendees.	Determine	CS						0%

Tucker et al., 2011 [33]	China, urban (LM).	2061 STI clinic attendees.	An immunochromatographic rapid treponemal test (Wantai, Beijing, China).	CS		Among those eligible, 81.6% agreed to syphilis rapid test.				

Yang et al., 2013 [44]	China, rural (LM).	27,150 ANC attendees.	Acon Biotech.	CS					73.6% of those diagnosed received treatment.	0.39%

CS: cross-sectional; CC: case control; CRT: clustered randomized trial; STPOC: simultaneous triple point-of-care testing; STAT: same day testing and treatment; ICS: immunochromatographic strip; RPR: rapid plasma reagin; TPHA: *Treponema  pallidum* particle agglutination assay;L: low-income economies; LM: low-middle income economies; UM: upper-middle income economies; H: high-income economies [56]; STD: sexually transmitted diseases; FSW: female sex workers; ANC: antenatal clinic; MSM: men who have sex with men; TG: transgender; NR: not reported being member of a risk group; MV: mobile van; STI: sexually transmitted infections; MSW: male sex workers; IDU: injecting drug users.
